# How do perceptions of information usefulness and green trust influence intentions toward eco-friendly purchases in a social media context?

**DOI:** 10.3389/fpsyg.2024.1429454

**Published:** 2024-08-02

**Authors:** Meifen Wu, Ruyin Long

**Affiliations:** ^1^School of Economics and Management, China University of Mining and Technology, Xuzhou, China; ^2^Fenner School of Environment and Society, Australian National University, Canberra, ACT, Australia; ^3^School of Business, Jiangnan University, Wuxi, China

**Keywords:** marketing on social media, green trust, perceptions of information usefulness, eco-friendly purchase intention, SOR theory

## Abstract

Drawing upon the stimulus-organism-response framework and incorporating green trust and perceptions of information usefulness, we formulated a model to explore how marketing on social media impacts consumers’ intentions towards eco-friendly purchases, using eastern Chinese cities as a case study. The findings indicate that: (1) marketing on social media significantly boosts intentions for eco-friendly purchases, and green trust positively affects the perceptions of information usefulness. (2) Green trust and perceptions of information usefulness jointly act as mediators between social media marketing and eco-friendly purchase intentions, with green trust exhibiting a stronger effect (0.306 > 0.122). The multi-group analysis findings indicate significant disparities in several potential pathways as a result of moderating factors such as educational attainment, etc. The benefits are especially apparent in women, people with middle to high incomes, people with intermediate to high levels of education, and people who engage with social media for over three hours per day. Through the effect analysis between marketing on social media, green trust, and perceptions of information usefulness on consumers’ intentions towards eco-friendly purchases, this study offers insights to social media platforms, businesses, and policymakers, enabling them to enhance strategies for fostering eco-friendly consumer behavior through social media channels.

## 1 Introduction

With pollution and climate change becoming the concerns of global society, people are increasingly interested in green products (Zhang et al., 2018). Due to social media’s influence on consumers’ purchasing behavior, utilizing social media for marketing products or services has grown tremendously in recent years (Park et al., 2021). To foster sustainable consumption behavior, it is crucial to scrutinize consumers’ inclinations to purchase eco-friendly products while using social media.

According to Sun et al. (2018), the eco-friendly product itself and its production process are resource-saving, safe and recyclable. Consumers’ willingness to make eco-friendly efforts can also be defined as their tendency to buy eco-friendly products, according to Gao et al. (2016). For how to promote consumers’ green purchasing behavior and willingness, scholars’ research is mainly carried out from two aspects: internal characteristics and external situational factors of consumers. The intrinsic idiosyncratic factors affecting green purchases include demographic factors (Lee, 2009) and psychological variables such as environmental values, environmental knowledge, environmental responsibility, environmental attitude, environmental concern, perceived behavioral effectiveness, consumer innovation, and lifestyle (Wang et al., 2010; Lao, 2013; Sheng et al., 2017). The external situational factors that affect consumers’ green purchases include product price, convenience of use, availability of information, social norms, etc. These factors affect consumers’ willingness to buy green by putting pressure on them or limiting their ability to purchase (Della Lucia et al., 2007; Wang and Wang, 2011). However, current research indicates the necessity for a more comprehensive comprehension of potential consumers’ intentions to obtain eco-friendly products via social media to further progress in this domain.

As a media with a wide audience, according to Huang (2016), social media provides users with more possibilities to spread and obtain information, which is vital to promoting individuals’ willingness and attitude to buy eco-friendly products. Therefore, social media platforms have been utilized by more corporations to promote products and develop new users (Sun and Wang, 2020), which is called marketing on social media. The related literature of marketing on social media has analyzed it in different contexts. Taking Sun et al. (2022) as an example, the effect of marketing on social media on the attitude and purchasing behavior about eco-friendly products was analyzed in the post-epidemic era. Different from previous studies, we aim to further analyze how perceptions of information usefulness and green trust influence eco-friendly purchase intention in the social media context. Firstly, as Holdack et al. (2022) stated, perceived information is a vital factor that affect individual’ shopping decisions in virtual reality. The existing literature (e.g., Alalwan, 2018) supports that information perception is the reason for determining the effective completion of individuals’ buying targets and offering guarantees for them. Secondly, as Lewandowska et al. (2017) mentioned in their study, consumer trust and engagement are important for green marketing as a sustainable marketing strategy, mainly because the crisis of trust is a major issue in green marketing, which is manifested in a lack of trust during the process of ecological information dissemination (Lemke and Luzio, 2014) as well as individuals’ own greenwashing experiences (Du, 2015). It has been verified in related literature about green marketing that green trust has an impact on eco-friendly behavior. However, the empirical results were not enough to demonstrate whether it is perception of information usefulness that has an impact on green trust or vice versa, and the direction of the correlation between the two is not clear. Some literature, for example, the process leading to forming eco-friendly behavior was described by Trivedi et al. (2018), which includes the effect of media and individuals’ attitudes towards eco-friendly wrapping, coupled with the efficiency of ecological concern and consumer perception. These studies on social media and eco-friendly purchase intention or decision have not comprehensively considered the influence mechanism of intentions towards eco-friendly purchasing in the effect of perceptions of information usefulness and green trust in eastern China and the comparative analysis of different demographic variables and social media use time among different groups.

Overall, a theoretical model was put forward derived from the Stimulus-Organism-Response (SOR) model and the Technology Acceptance Model (TAM). SOR is utilized for understanding the relationship between people’s received stimulus (S) and their internal evaluation (O) and response (R). This framework has been used in advertising (Olney et al., 1991). It is believed that stimuli, or outside influences, affect the internal state of individuals, or organisms, which then influence the attitude and behavior of individuals or reactions. Stimulation can have positive or negative effects. Individuals’ emotional and cognitive responses describe internal processes (Chan et al., 2017). As Alalwan (2018) and Kang et al. (2020) verified in their study the perceptions of information usefulness reflect one’s favorable judgment on advertising, reduce invasiveness, and encourage favorable marketing outcomes. Therefore, it can be preliminarily assumed that consumers’ green trust and perceptions of information usefulness will influence marketing on social media and eco-friendly purchase intentions.

This study seeks to investigate the following issues: (1) What is the arrow of the relationship between perceptions of information usefulness and green trust? Do they all play the role of intermediary variables in the model, and if so, which one plays a greater role? (2) What is the correlation between the variables proposed in the SOR model and eco-friendly purchase intention? The innovations and contributions of this study are: (1) This study explores the complex processes by which marketing on social media influences intentions toward eco-friendly purchases in the social media context, and it is based on the SOR theory and TAM. It contributes to the SOR theory by highlighting the essential functions that perceptions of information usefulness and green trust play as important antecedent factors. (2) The mechanisms of the effects of marketing social media on intentions towards eco-friendly purchases have expanded with the addition of green trust and perceptions of information usefulness as chain mediating variables. This has also given rise to a fresh viewpoint for explaining the discrepancies in previous research. In addition, the direction and effect values of the correlation between perceptions of information usefulness and green trust are clarified.

The other parts of this study are structured as follows: a related literature review is conducted and research hypotheses focusing on the components of the SOR model and inter-variable relationships are established in Section 2. Sample collection, research methods, and process are shown in Section 3. In Section 4, numerical research findings are presented. Section 5 provides a conclusion and discussion. Lastly, Section 6 summarizes the limitations and further directions.

## 2 Literature review and research hypothesis

### 2.1 SOR theory

The SOR model originally came from the discipline of environmental psychology, which was used to clarify that the effect of the environment on human behavior was produced by human psychology. The model was put forward by Mehrabian and Russell in 1974. It indicates that the environment is a stimulating factor that affects individuals, and these stimulating elements are described as attributes that can influence the perceptions of individuals (Su and Swanson, 2017). These stimulating factors (S) will lead to changes in consumers’ cognitive or emotional state (O). This kind of change mainly refers to the conscious or unconscious psychological state of the body after being stimulated. In essence, it is an internal state of the individual composed of “perception, physiology, feeling, and thinking activities” (Bagozzi, 1986), and the cognitive organism further affects the behavior of consumers after receiving stimulation (R).

In previous studies, the SOR model was often used to study consumer behavior. For example, trust was regarded as an inner condition parameter and the connection involving electronic services and intended actions (Gupta et al., 2019). Marketing on social media can serve as an external stimulus, and the stimulus factors are related to the info about eco-friendly products, such as energy-saving certification. Organism refers to the internal characteristic factors of individuals, including cognitive factors and emotional factors (Bagozzi, 1986). As an external stimulus, marketing on social media will trigger individual psychological and physiological changes in consumers’ cognition and emotions. Cognitive changes include all changes related to information acquisition, processing, retention, and retrieval by consumers. Emotional changes reflect the excitement and pleasure of individuals under external stimuli. In this study, this emotional response is the emotion and feeling aroused by marketing on social media. Response refers to the consumer’s behavioral response, such as the purchase intention (Batra and Ray, 1986). Consumers may have some kind of purchasing behavior after complicated physiological and psychological reactions. In the SOR framework, green trust and perceptions of information usefulness have been accepted by some scholars as internal state variables of the organism (e.g., Ahmad and Zhang, 2020; Zafar et al., 2023). Therefore, in this study, green trust and perceptions of information usefulness can be selected as the internal evaluation variables of consumers in the SOR framework. Applying the SOR model, marketing on social media (S) stimulates consumers’ green trust in green products and their internal evaluation of perceptions of information usefulness (O), ultimately producing the buying inclination of individuals (R).

### 2.2 Marketing on social media and eco-friendly purchase intention

As Steinhoff et al. (2019) stated, marketing on social media uses the operation mode of online relationships. Alam et al. (2023) analyzed through an experimental survey whether social media affects individuals’ eco-friendly buying intentions and the conclusions indicated that social media is crucial in shaping behavioral choices such as intentions as consumers make decisions. The importance of marketers using e-marketing strategies to disseminate details regarding environmentally sustainable products on social platforms was also emphasized. In addition, as Zahid et al. (2018) stated, social media raises consumers’ environmental awareness, which can promote individuals’ awareness and search for information related to adopting a green lifestyle and influence intentions to purchase. Nekmahmud et al. (2022) studied social media’s effects on changing intentions for eco-friendly purchases of individuals, through which it was concluded that marketing on social media contributes positively to eco-friendly buying intentions and sustainable consumption behavior. Similarly, Wu and Long (2024) analyzed the impact of environmentally conscious messaging on the intention toward eco-friendly consumption of social media users in their study, regarded marketing on social media as a green communication way, and empirically analyzed to get the conclusion that marketing on social media improves the sustainability-oriented purchasing inclination. Therefore, this study assumes that:

*H1*. Marketing on social media positively impacts eco-friendly purchase inclination.

### 2.3 The mediating influence of green trust

Green trust is defined as the beliefs and prospects of individuals originating from the ability, trustworthiness, and positive image of eco-friendly product manufacturers, and the resulting willingness to trust the enterprises and products. Consumers typically exhibit greater trust in the company’s products if they are promoted as eco-friendly (Kalafatis et al., 1999). Chen and Chang (2012) applied structural equation modeling to verify the intermediary function of green trust when researching intentions for eco-friendly purchases. Schlosser et al. (2006) used an experimental method to study the inclination to purchase online of individuals and concluded that consumers’ trust can have a substantial impact on their inclination to purchase online. Bateman and Valentine (2015) stated in their study that consumers’ trust in salespeople positively affects their purchase intention when studying the sales interaction process between salespeople and consumers. To determine the readiness of consumers to purchase eco-friendly appliances, Asif et al. (2023) concluded that green trust positively impacts the inclination to buy energy-saving appliances through an empirical study of Pakistani consumers. According to Rousseau et al. (1998), trust directly influences consumers’ propensity to buy. The trust of consumers in eco-friendly items affects their purchasing choices based on how much they trust them (Garbarino and Johnson, 1999). This study assumes that:

*H2*. Green trust performs the function of a mediating factor in the connection involving marketing on social media and the intentions toward purchasing eco-friendly products.

### 2.4 The intermediary function of perceptions of information usefulness

Marketing on social media can be viewed, using the SOR model, as an external stimulus that affects consumers’ perceptions of the information usefulness of eco-friendly products. Marketing on social media is only successful as an external stimulant when customers believe the content is really powerful. Perceptions regarding the utility of information about how consumers gauge the efficacy of the information conveyed in advertisements in aiding their decision-making process (Matthes and Wonneberger, 2014), which is regarded as a cognitive state in SOR (Goi et al., 2014). Research indicates that customers’ need for product characteristic information is satisfied by green advertising, which helps them make more informed judgments about what to buy. Information with a high information utility is seen as dependable and helpful. Conversely, poor information usefulness denotes the belief that information is false and disregarded (Wei et al., 2017). Regardless of whether a product has market value, consumers’ cognitive responses to advertising influence their intention to buy, according to research on green advertising that supports the SOR model. Similarly, within the framework of this study, individuals’ perceptions of information usefulness presented in green advertisements determine their inclinations towards eco-friendly purchasing. Consequently, the following research hypothesis is presented:

*H3*. Perceptions of the usefulness of information serve as a mediating factor in the correlation between marketing on social media and the intentions toward purchasing eco-friendly products.

### 2.5 The chain mediating role of perceptions of information usefulness and green trust

The concept of green trust was originally put forward against the background of green consumption. People’s psychological states are referred to as trust, and they are willing to accept vulnerability if it results in positive experiences (Chen and Chang, 2013). According to Flavian et al. (2005), trust can lower customers’ risk perceptions, which will increase the informational value of associated green brands or products and encourage consumer activity. Consumers’ trust in green products will affect their subjective judgment of product value. If consumers believe that an enterprise can produce green products and think that it is reliable and well-intentioned, it will have a more positive evaluation of the value of its items. Xie et al. (2019) show that reference groups influence perceived value through green trust and then influence sustainability-oriented purchase intent.

In this study, rational behavior theory and TAM (Davis, 1989) are used to explain the effectiveness of green trust and perceived information. The cornerstone of rational action is the main belief or perception that determines attitude, intention, and behavior (Pavlou, 2003). TAM holds that action inclination determines the system to use, and action inclination is decided by attitude toward using and perceptions of usefulness. Saura et al. (2022) predicted that there existed a robust positive association between perceptions of usefulness and trust of individuals. Sterrett et al. (2019) investigated the effect of the trust of sharers (public figures) on credibility and participation. Social media users believe more in news or information shared with public figures who are more willing to share their trust (Majerczak and Strzelecki, 2022). When Liu and Chen (2023) studied the deeply forged information, it was confirmed that because the characters and languages involved in the deeply forged information were all real and only replaced or spliced, users would rely more on the source clues. As the indirect source of the deeply forged information, whether users trusted the sharer would also affect their perception and behavior toward the information. Therefore, the following research hypothesis is proposed:

*H4*. Green trust and perceptions of information usefulness collectively serve as intermediary factors in the correlation involving marketing on social media and intentions toward purchasing eco-friendly products.

**Figure 1:**
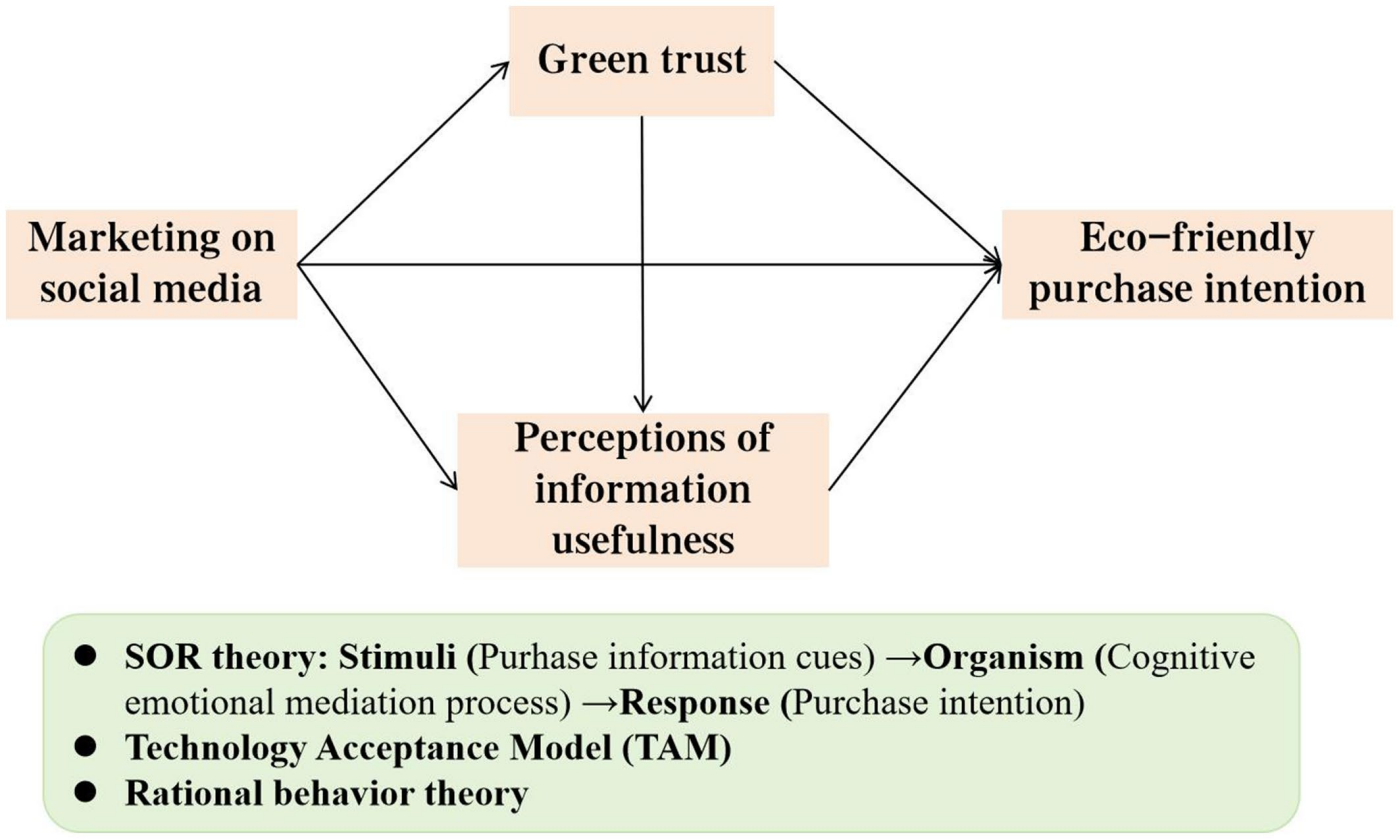
Theoretical model.

To sum up, Figure 1 shows the theoretical model.

## 3 Data collection and research methodologies

### 3.1 Sample collection

The survey was conducted with social media users in the eastern region, which was chosen mainly because it is a densely urbanized, geographically distributed region with a wide range of geographical areas, densely populated, economically developed, and with high consumption of resources by its residents. Four main economic areas can be divided in China, including the east, middle, northeast, and west. The eastern part of China includes provinces or regions like Beijing, Tianjin, Hebei, Shanghai, Jiangsu, Zhejiang, Fujian, Shandong, Guangdong, and Hainan, and it is a relatively developed economic zone and covers a wide area in geographical distribution. Wenjuanxing was used to conduct the survey, and the purpose of the questionnaire was clearly explained and the significance of truthful completion was emphasized before the questionnaire was started. In addition, definitions and examples were given before the questionnaire started to make the definition of eco-friendly products clearer to the subjects. The demographic section of the questionnaire collected information about respondents’ age, gender, etc. (as depicted in Table 1), and a 7-point Likert scale was conducted to test the perceptions and attitudes of respondents toward marketing on social media, green trust, perceptions of information usefulness, and eco-friendly purchase intention. The survey process includes two pre-surveys and a formal survey. Although the scales are all from authoritative scales published in the past, the reliability and validity of the questionnaire were not good in the first pre-survey (534 valid questionnaires were distributed), so a new round of pre-survey was carried out (227 valid questionnaires were distributed). This pre-survey showed that the reliability and validity of each variable scale met the requirements, and then a formal survey was carried out. Through the process of revising the scale in the pre-survey and screening invalid samples in the formal survey, 686 valid questionnaires were finally obtained.

**Table 1:** Demographic information of subjects.

Variable		Frequency	Percentage
Gender	Male	355	51.7%
	Female	331	48.3%
Age	Below 20	113	16.5%
	21–30	281	41.0%
	31–40	210	30.6%
	41–50	53	7.7%
	51 and over	29	4.2%
Education	Junior high school or below	15	2.2%
	High school and technical secondary school	48	7.0%
	Junior college and higher vocational education	153	22.3%
	Bachelor’s degree	416	60.6%
	Master’s degree or higher	54	7.9%
Income	≤2000	104	15.2%
	2001–5,000	117	17.1%
	5,001–8,000	162	23.6%
	8,001–12,000	174	25.4%
	12,001–17,000	84	12.2%
	>17,000	45	6.6%

### 3.2 Questionnaire design and analytical methods

**Figure 2:**
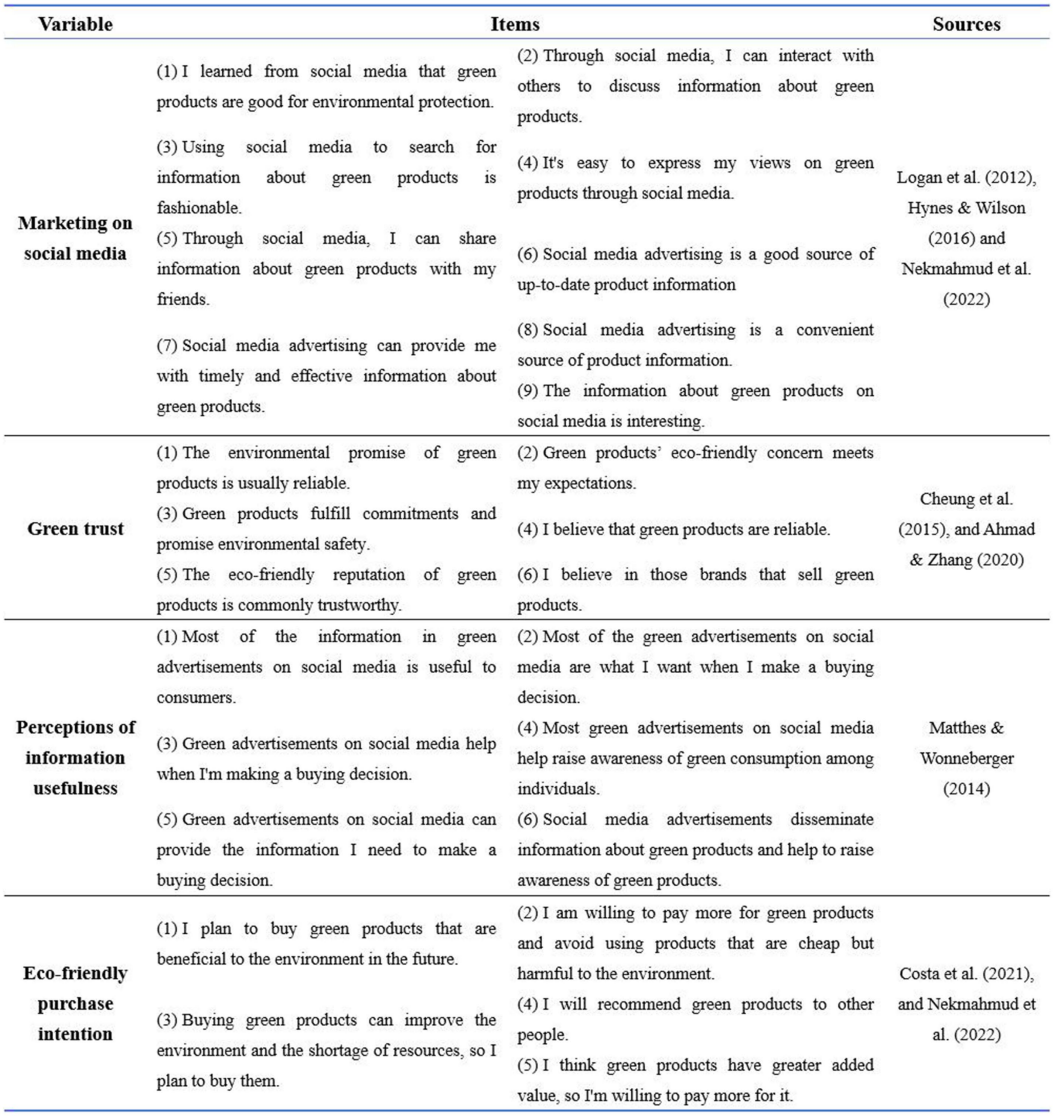
The detailed scale items and sources of variables.

Every variable’s scale is drawn from the authorized scale and suitably adjusted based on the research context. The design process of marketing on social media, perceptions of information usefulness, green trust, and eco-friendly product purchasing intentions are shown in Figure 2.

To test to proposed theoretical model, some methods and processes were conducted. Firstly, to verify the validity and reliability of the questionnaire, Cronbach’s α and confirmatory factor analysis (CFA) are employed. Secondly, the Structural Equation Model (SEM) (Anderson and Gerbing, 1988) was utilized in this study. The study assessed the correlation among marketing on social media, perceptions of information usefulness, green trust, and intentions toward eco-friendly purchases with the assistance of AMOS 22.0. Thirdly, Hayes SPSS Process Macro (Hayes, 2013) was used to test the mediating effect. We used the Bootstrapping approach to examine the direct effects and the intermediary functions of the perceptions of information usefulness and green trust between marketing on social media and intentions to make eco-friendly purchases. Each proposed path was examined for the impact of moderating variables using an analysis of multi-group comparative.

## 4 Research results

### 4.1 Analysis of reliability and validity

As depicted in Table 2, it is clear that Cronbach’s α of marketing on social media, green trust, perceptions of information usefulness, and intentions toward eco-friendly purchase all exceed 0.85, which means all scales have good reliability. A CFA of marketing on social media, green trust, perceptions of information usefulness, and intentions toward eco-friendly purchase were conducted to evaluate the validity of convergence and discriminant. All of the study’s items have standardized factor loads greater than 0.6, the SMC value is beyond 0.5, the CR value is beyond 0.85, and the AVE value is beyond 0.5, which demonstrates the strong convergence validity of the research scales (The criteria are shown in Table 3).

**Table 2:** Results and validity of confirmatory factor analysis for each research construct.

Constructs	Index	Estimation of model parameters				Convergent validity				Cronbach’s *α*
		Estimate	S.E.	C.R.	*p*	Estimate	SMC	CR	AVE	
MSM	MSM1	1.000				0.758	0.575	0.921	0.565	0.923
	MSM2	1.156	0.054	21.297	***	0.780	0.608			
	MSM3	1.084	0.054	20.126	***	0.744	0.554			
	MSM4	1.083	0.055	19.845	***	0.734	0.539			
	MSM5	1.108	0.054	20.377	***	0.751	0.564			
	MSM6	1.074	0.052	20.808	***	0.765	0.585			
	MSM7	1.095	0.053	20.773	***	0.761	0.579			
	MSM8	1.031	0.052	19.943	***	0.733	0.537			
	MSM9	1.144	0.058	19.887	***	0.736	0.542			
GTR	GTR1	1.000				0.835	0.697	0.925	0.672	0.924
	GTR2	1.018	0.037	27.243	***	0.841	0.707			
	GTR3	1.007	0.039	26.135	***	0.820	0.672			
	GTR4	0.903	0.038	23.986	***	0.775	0.601			
	GTR5	0.994	0.037	26.977	***	0.836	0.699			
	GTR6	1.008	0.039	25.612	***	0.809	0.654			
PIU	PIU1	1.000				0.818	0.669	0.902	0.605	0.902
	PIU2	1.028	0.040	25.950	***	0.833	0.694			
	PIU3	0.938	0.038	24.639	***	0.806	0.650			
	PIU4	0.994	0.046	21.769	***	0.733	0.537			
	PIU5	0.888	0.039	22.678	***	0.760	0.578			
	PIU6	0.798	0.039	20.536	***	0.710	0.504			
EPI	EPI1	1.000				0.814	0.663	0.883	0.601	0.895
	EPI2	0.967	0.037	26.377	***	0.758	0.575			
	EPI3	1.091	0.046	23.654	***	0.795	0.632			
	EPI4	1.053	0.048	22.086	***	0.756	0.572			
	EPI5	1.075	0.049	21.787	***	0.751	0.564			

*** signifies a significance level of *p* < 0.001, ** indicates a significance level of *p* < 0.01, and * denotes *p* < 0.05. MSM, marketing on social media; GTR, green trust; PIU, perceptions of information usefulness; EPI, eco-friendly purchase intention. The same is below.

**Table 3:** Ideal value of each index.

Index	Ideal value	References
SMC	>0.5	Hair et al. (2010)
CR	>0.7	
AVE	>0.5	
RMSEA	<0.08	Bollen (1989)
SRMR	<0.08	
GFI, TLI, IFL, CFI	>0.90	Kline (2015)
$\chi^{2}/$ df	1–5	

### 4.2 Test for homologous method deviation

To evaluate the varying validity within the variables, we assessed the model fitness of marketing on social media, green trust, perceptions of information usefulness, and intentions to make eco-friendly purchases. The research model has fitting indicators that are superior to those of the other three substitute models, as shown by the values of indicators like RMSEA and CFI in Table 4. This demonstrates that marketing on social media, green trust, perceptions of information usefulness, and eco-friendly purchase intention are distinct constructs, affirming the robust construct validity of the research scales.

**Table 4:** Results of discriminant validity testing between variables.

Model	$\chi^{2}/$ df	RMSEA	CFI	TLI	IFI	SRMR
Four-factor model (MSM, GTR, PIU, EPI)	2.871	0.052	0.961	0.956	0.961	0.0283
Unmeasured potential factor model	2.575	0.048	0.970	0.963	0.970	
Three-factor model (MSM, GTR + PIU, EPI)	3.666	0.062	0.944	0.937	0.944	
Two-factor model (MSM, GTR + PIU + EPI)	3.836	0.064	0.940	0.933	0.940	
One-factor model (MSM + GTR + PIU + EPI)	4.888	0.075	0.918	0.909	0.918	

### 4.3 Confirmatory factor analysis incorporating common method factors

Since marketing on social media, green trust, perceptions of information usefulness, and intentions to make eco-friendly purchases rely on self-reports from participants, the study draws data from a single pool of subjects, introducing the possibility of common method variance. To evaluate the extent of this variance, two approaches were employed. Firstly, employing the Harman single-factor method, the analysis revealed that the fit indices of the single-factor model were less than optimal: $\chi^{2}/$ df = 4.888, RMSEA = 0.075, CFI = 0.918, TLI = 0.909, and IFI = 0.918, implying that the common method variation was not severe to a certain extent. The unmeasurable factor method is employed in this study to examine common method variation risk because the Harman single-factor approach is not sufficiently responsive to do so. The idea behind this approach is to build a variation factor model using a common method and then compare if the model’s fitting index is superior to the four-factor model’s. Table 3 shows that the extent of enhancement of RMSEA and SRMR is limited to 0.05 when a common method variation factor is introduced to the four-factor model to create an unmeasurable potential factor model. The CFI, TFI, and other indices have improved by approximately 0.02. There is virtually little difference between the four-factor model and the standard method variation factor model. Consequently, it is possible to infer that there is no significant common technique bias in the measurement by integrating the analytical results of these two approaches.

### 4.4 Mean, standard deviation, and correlation coefficients of the variables

**Figure 3:**
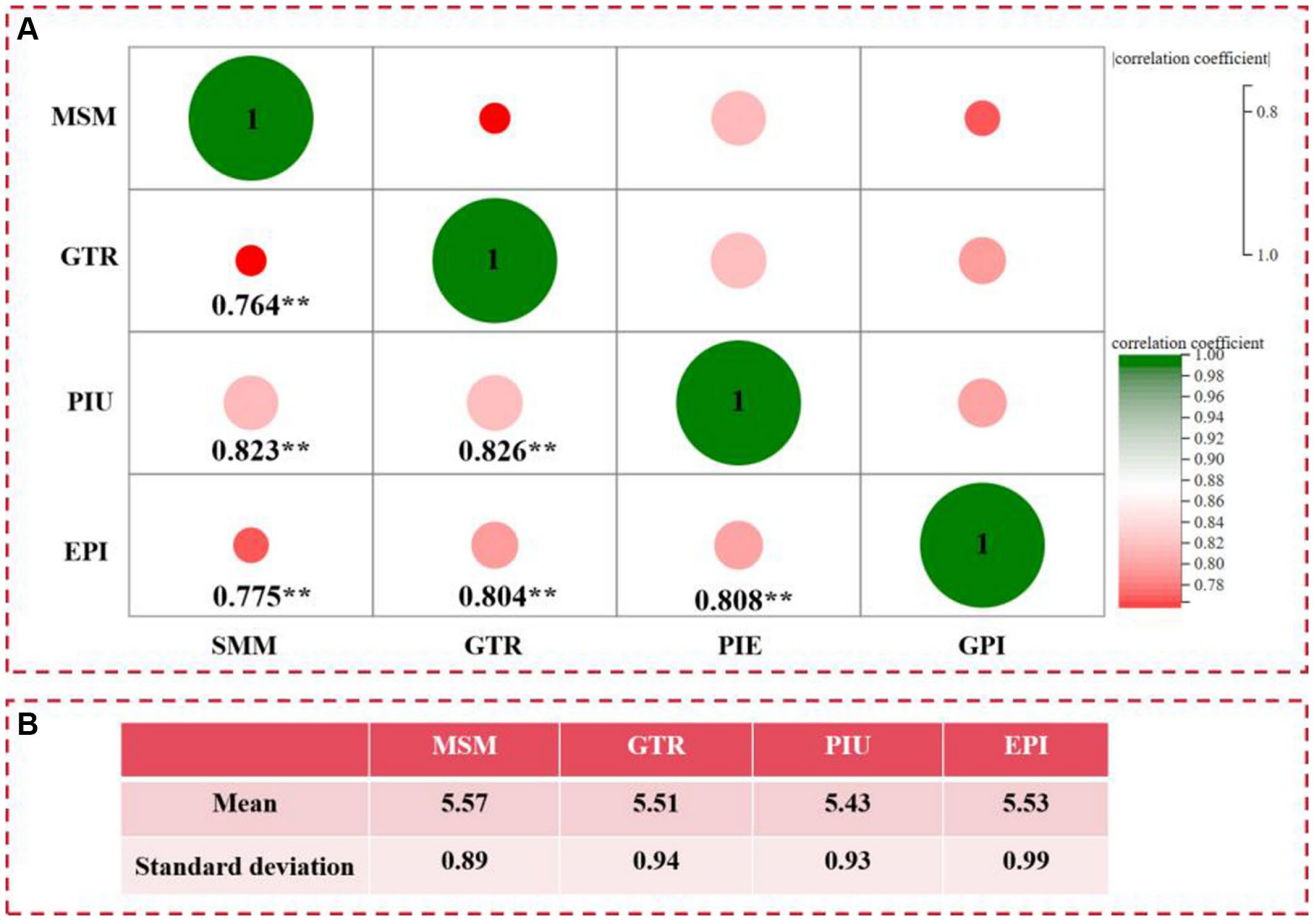
Mean, standard deviation, and correlation coefficients of the variables. **(A)** Correlation of variables. **(B)** Mean and standard variance of each variable.

Marketing on social media, green trust, perceptions of information usefulness, and eco-friendly purchase intention are analyzed by descriptive analysis and Pearson Correlation Analysis. As depicted in Figure 3, the correlation coefficients between the variables are less than 0.9, implying that the data is acceptable.

### 4.5 Hypothesis test

#### 4.5.1 Test of main effect and mediating effect

The main effect and intermediate effect are tested using the hierarchical regression method, and the findings are illustrated in Table 5. The main effect is confirmed first. Model 6 extends Model 5 by using marketing on social media. Model 5 is the regression model of control factors for eco-friendly purchase intention. H1 is confirmed by the findings, which demonstrate that marketing on social media significantly increases the intentions toward eco-friendly purchases of individuals (*r* = 0.744, *p* < 0.001). Second, it is confirmed that perceptions of information usefulness and green trust have an intermediary function. The consequence of social media marketing on the perceptions of information usefulness and green trust is examined through models 2 and 4, respectively. The findings support the validity of H2 and H4, demonstrating that marketing on social media significantly improves perceptions of information usefulness and green trust (*r* = 0.750, *p* < 0.001; *R* = 0.814, *p* < 0.001). By incorporating green trust into Model 6, Model 7 demonstrates that intentions toward eco-friendly purchases are positively impacted by green trust (*r* = 0.496, *p* < 0.001). Model 7’s marketing on social media coefficient on eco-friendly purchase intention drops from 0.744 to 0.373 when compared to model 6, suggesting that green trust may act as a partial mediating factor between marketing on social media and intentions toward eco-friendly purchase. H3 is also examined. Perceptions of information usefulness are added to Model 6 in Model 8, and the results illustrate a substantial beneficial effect on the intentions toward eco-friendly purchasing (*r* = 0.517, *p* < 0.001). Model 8’s marketing on social media coefficient on eco-friendly purchase intention decreases from 0.744 to 0.324 when compared to model 6, suggesting that perceptions of information usefulness partially mediate the connection between marketing on social media and eco-friendly purchase intention. This finding supports hypothesis H5.

**Table 5:** Results of the main effect and intervening effect tests.

Variables	Green trust		Perceptions of information usefulness		Eco-friendly purchase intention			
	Model 1	Model 2	Model 3	Model 4	Model 5	Model 6	Model 7	Model 8
Gender	0.034	0.004	0.084*	0.052*	0.060	0.030	0.028	0.004
Age	0.062	0.030	0.059	0.024	0.089*	0.056*	0.042	0.044
Education	0.133**	0.009	0.144***	0.010	0.196***	0.073**	0.068**	0.068**
Income	0.126**	0.045	0.106*	0.019	0.128**	0.048	0.026	0.038
Marketing on social media		0.750***		0.814***		0.744***	0.373***	0.324***
Green trust							0.496***	
Perceptions of information usefulness								0.517***
*R* ^2^	0.058	0.587	0.059	0.682	0.096	0.617	0.718	0.702
Δ*R*^2^	0.053	0.584	0.053	0.679	0.091	0.614	0.716	0.699
*F*	10.554	193.518	10.596	291.061	18.126	219.079	288.661	266.572

#### 4.5.2 Chain mediating effect test

The chain mediating roles of green trust and perceptions of information usefulness were assessed using the Process tool in SPSS, with the findings presented in Table 6. In the path “marketing on social media → green trust → intentions toward eco-friendly purchasing,” the interval with 95% confidence is [0.185, 0.420], excluding 0, suggesting that the intervening effect is statistically substantial and H3 is supported. In the path “marketing on social media → perceptions of information usefulness → intentions toward eco-friendly purchasing,” the interval with 95% confidence is [0.062, 0.220], excluding 0, suggesting that the intervening effect is statistically substantial and H5 is supported. In the path “marketing on social media → green trust → perceptions of information usefulness → intentions toward eco-friendly purchasing,” the interval with 95% confidence is [0.097, 0.246], excluding 0, indicating that green trust and perceptions of information usefulness play a chain intermediary role involving marketing on social media and eco-friendly purchase intention, and H6 is supported.

**Table 6:** Test results of chain intermediary effect.

Effect path	Effect	Boot SE	BootLLCI	BootULCI	Proportion of total effect
Total indirect effect	0.583	0.064	0.448	0.693	68.18%
Ind1: marketing on social media → green trust → eco-friendly purchase intention	0.306	0.060	0.185	0.420	35.84%
Ind2: marketing on social media → perceptions of information usefulness → eco-friendly purchase intention	0.122	0.039	0.062	0.220	14.22%
Ind3: marketing on social media → green trust → perceptions of information usefulness → eco-friendly purchase intention	0.155	0.036	0.097	0.246	18.12%

### 4.6 Multi-group comparative analysis

This study utilizes multi-group analysis to examine whether the eco-friendly purchase intentions among different consumer segments conform to the proposed hypothetical model, examining the presence of parameter invariance. Demographic variables and daily time spent on social media were employed as moderator variables in the multi-group analysis conducted. The CFI, TLI, and IFI values of the model varied from 0.903 to 0.927, all greater than 0.90. RMSEA values spanned from 0.051 to 0.055, all less than 0.08. The significance level of the chi-square statistic did not reach the significance level. The above index values show that the multi-group analysis model is well adapted to the sample data.

**Figure 4:**
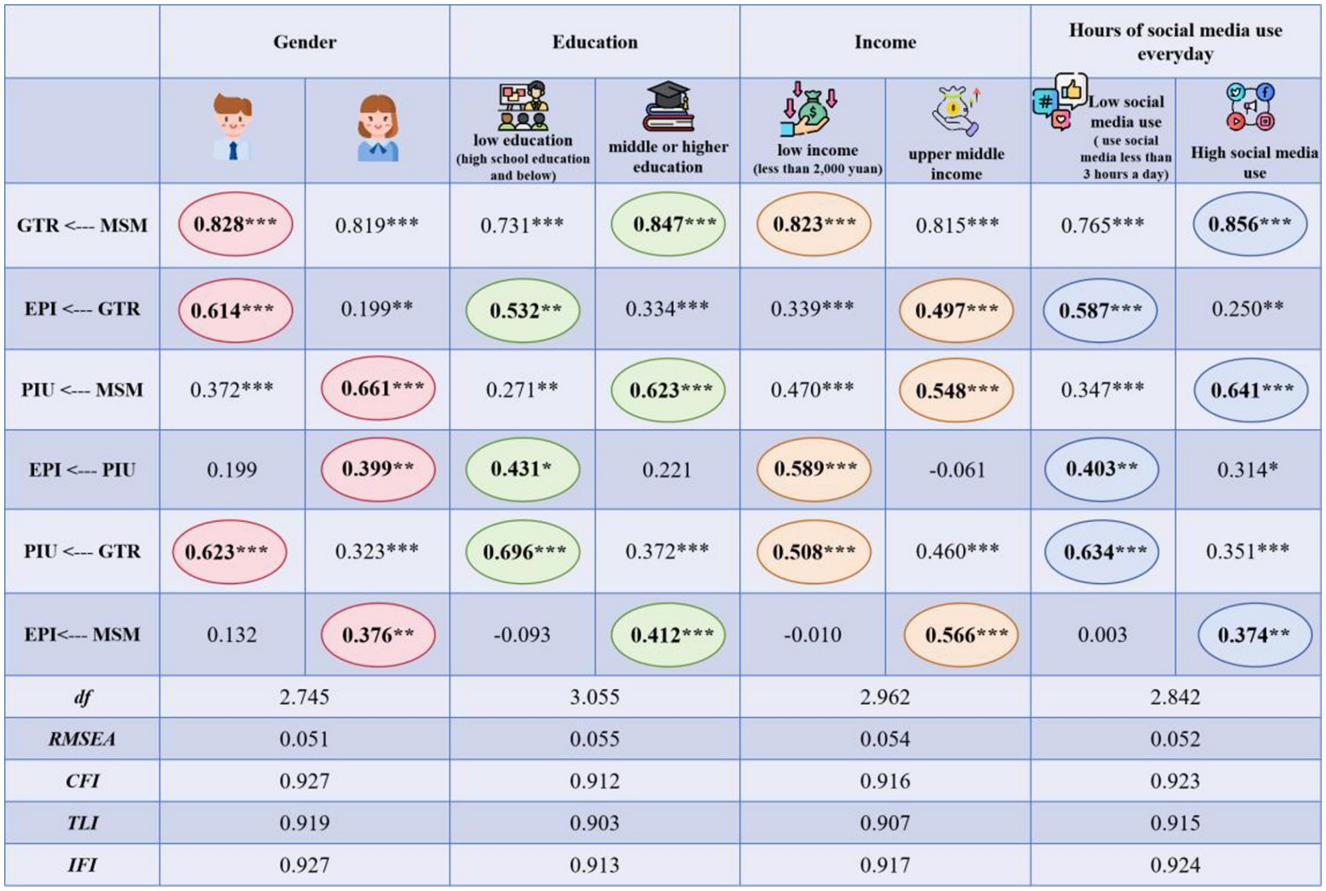
Multiple-group analysis results. The criteria for dividing groups refer to Wu and Long (2024).

As depicted in Figure 4: (1) in the pathway where marketing on social media positively influences green trust, men exhibit greater significance compared to women (*β* = 0.828, *p* < 0.001). The influence of middle and high education is more significant than that of low education (*β* = 0.847, *p* < 0.001). Among the low-income groups, the high-income group has a greater influence (*β* = 0.823, *p* < 0.001). Individuals who engage with social media for over 3 h have more significant influence than those who spend fewer than 3 h on social media (*β* = 0.856, *p* < 0.001). After summarizing the findings of the aforementioned analysis, this study found that among men, those with greater incomes but lower levels of education, and those who dedicate more than 3 hours daily to social media, marketing on social media dramatically increases green trust. The reason for this result may be that using social media for more than 3 hours has given individuals more opportunities to get in touch with information related to green products. In addition, this kind of person has a higher degree of education, so their understanding of this information is relatively high, which in turn affects their confidence in green products and thus their stronger trust in green products.

(2) Concerning the effect of green trust on the intentions toward purchasing eco-friendly items, men (*β* = 0.614, *p* < 0.001), low education (*β* = 0.532, *p* < 0.01), middle and high income (*β* = 0.497, *p* < 0.001) and low social media contact (*β* = 0.587, *p* < 0.001).

(3) Upon analyzing how social media marketing influences perceptions of information usefulness, compared with men, women have a more significant influence (*β* = 0.661, *p* < 0.001). Individuals’ effects in the middle and high education (*β* = 0.623, *p* < 0.001) and the importance of the middle and high-income brackets are notable (*β* = 0.548, *p* < 0.001). Furthermore, the effect of people individuals who spend over 3 h on social media is more substantial (*β* = 0.641, *p* < 0.001). This study believes that this is because women with middle and high education and income possess a greater sense of societal obligation, information understanding, and attention to environmental development. They also have high contact with social media (the average daily use time is more than 3 h), so their perception of information such as green products is more effective.

(4) Regarding the effect of perceptions of information usefulness on eco-friendly purchase intention, women (*β* = 0.399, *p* < 0.01), low education (*β* = 0.431, *p* < 0.05), low income (*β* = 0.589, *p* < 0.001) and low contact with social media (*β* = 0.403, *p* < 0.01). However, the influence of males, individuals with middle to high education levels, and those in the middle to high-income groups is not statistically meaningful.

(5) From the impact of green trust on perceptions of information usefulness, men (*β* = 0.623, *p* < 0.001), low education (*β* = 0.696, *p* < 0.001), low income (*β* = 0.508, *p* < 0.001) and low social media contact (*β* = 0.634, *p* < 0.001).

(6) Regarding the impact of social media marketing on intentions to purchase eco-friendly items, women (*β* = 0.376, *p* < 0.01), middle and high education (*β* = 0.412, *p* < 0.001), middle and high income (*β* = 0.566, *p* < 0.001) and high exposure to social media (*β* = 0.374, *p* < 0.01). Men, low education, low income, and low social media contact groups have no significance at all.

Combining the two paths of marketing on social media → green trust, green trust → eco-friendly purchase intention and marketing on social media → eco-friendly purchase intention, it can be seen that women, people with medium and Individuals with higher education, middle to high-income levels, and extensive exposure to social media all exhibit significant influence across these three pathways. However, for males, individuals with lower education and income levels, and those with limited exposure to social media, although social media marketing significantly impacts both green trust and intentions to make eco-friendly purchases, its effect is particularly pronounced on eco-friendly purchase intentions. On the one hand, it shows that the path of transforming marketing on social media into eco-friendly purchase intention is impacted by many variables, among which green trust plays an important role, gender differences, the improvement of educational level, the increase in income, and the length of social media usage help to promote consumers’ eco-friendly purchase intention. Conversely, women tend to prioritize green products, particularly those with higher academic qualifications, while individuals in middle- and high-income brackets focus more on enhancing quality of life and contributing to social development. Moreover, frequent users of social media demonstrate greater receptivity and understanding towards information regarding green products, resulting in a higher inclination to purchase them. When consumers buy green products, they need to have the willingness to buy green subjectively and have enough information to support their confidence objectively. If consumers have difficulty understanding green products, do not believe in green products, or know little about green products, they find it challenging to form their eco-friendly purchase intention.

## 5 Conclusion and discussion

The main objective of this study is to evaluate consumers’ purchase intentions and observe how social media marketing positively affects consumers’ purchases of green products. The SOR theory and TAM were expanded through additional variables (green trust and perceived information utility), a theoretical framework for evaluating the green purchase intention of urban residents in eastern China through social media was developed, and an empirical test based on 686 consumer survey data in eastern China was made. The research results indicate that:

(1) Marketing on social media contributes considerably to the improvement of eco-friendly purchase intention. This shows that when focusing on marketing on social media, consumers will increase their awareness of green products and get more information about green products, thus increasing the individual’s eco-friendly purchase intention. Consistent with Alam et al. (2023), intentions toward eco-friendly purchasing were affected by social media, and social media plays a crucial role in forming the choices of individuals. Similarly, Zahid et al. (2018) revealed that social media significantly contributes to individual behavior and affects the intentions of buying. Trivedi et al. (2018) proposed that the increasing attention of the media to highlighting different environmental issues indeed forms a higher concern of consumers for ecology. To raise ecological consciousness and strengthen the attitude of citizens concerned about protecting environmental resources, some targeted activities can be used by non-profit organizations.

(2) Green trust and perceptions of information usefulness act as an intermediary in correlation involving marketing on social media and intentions of eco-friendly purchasing, respectively. It has been found that green trust is a key determinant of intentions for eco-friendly purchasing. The intermediary role of green trust has also been demonstrated in previous studies on green consumption behavior (e.g., Saif et al., 2024). We also concluded that marketing on social media benefits consumers’ eco-friendly purchase intention, either directly or indirectly through green trust, which correlates with the findings of earlier research. Several e-commerce literature show the effect of trust on buying intention, taking Jadil et al. (2022) as an example, they analyzed the impact of online trust on Morocco’s willingness to purchase online through an online survey and concluded that online trust significantly enhances the intention to purchase. Moslehpour et al. (2022) examined the effect of trustworthiness and brand perception as mediating variables involving marketing activities on social media and intentions of purchasing in the Indonesian context, where marketing activities on social media were divided into 5 aspects. Regarding the study of perceived information utility involving social media and eco-friendly buying intention, Luo et al. (2020) analyzed and concluded that perceived information utility plays a mediating role between skepticism of green advertisements across social platforms and eco-friendly buying intent. Similarly, social media marketing and individual skepticism toward green advertisements are essentially access to or attitudes toward info related to environmentally friendly products, both of which can influence consumers’ intention of eco-friendly purchasing through the mediating variable of perceived informational utility, which impacts consumers’ intentions of eco-friendly purchasing. Analyzing theoretically, the perception of information usefulness is an organism that will affect the consequences of consumers’ purchases. The SOR model’s supporting literature maintains that consumers’ purchase intentions are influenced by their cognitive responses to advertisements, independent of the product’s market value (Batra and Ray, 1986). Consumers’ intention of eco-friendly purchasing is related to the perceptions of information usefulness of social media, and higher information utility is associated with stronger purchase intention. Customers might not know enough about green products because of the knowledge imbalance that exists between businesses and customers (Matthes and Wonneberger, 2014). As a result, to engage in green consumerism, they would proactively search for information on environmentally friendly products.

(3) Green trust and perceptions of information usefulness act as a link mediating role between marketing on social media and intentions of eco-friendly purchasing. Green trust benefits perceived information utility, and the intermediary role of green trust is greater. Pittman and Abell (2021) show that a lower popularity index seems to be beneficial to green influencers, and an increase in trust in green influencers with lower popularity will enhance their attitude towards sponsored products and increase their willingness to buy. Consumers use information from green marketing mostly to determine whether or not to purchase eco-friendly items. Customers show more interest in eco-friendly products, browse, purchase, and use more eco-friendly items, and develop a greater level of trust in eco-friendly items when they observe the info given in green marketing as beneficial. Consumers think that the information presented in green advertisements is deemed more valuable. Thus, as Matthes and Wonneberger (2014) stated if the info is beneficial for customers, the credibility of eco-friendly assertions will be favorably evaluated. Conversely, people would typically utilize fewer environmentally friendly products when the informational usefulness is low. As Liao et al. (2019) mentioned in their research, when users have a high degree of trust in promoting their brands on social media platforms, they will demonstrate an elevated degree of belief in buying their products. Social information tends to spread rapidly among customers. When consumers think this information is useful, it will increase their trust and confidence in products, thus affecting their readiness to purchase eco-friendly items and minimizing the risk of buying needed products or services.

(4) The multi-group analysis results indicate meaningful variations in moderating factors like gender, level of education, and time spent on social media across different hypothetical pathways. Some studies have confirmed that demographic variables affect eco-friendly purchase intentions and that there are differences across groups (e.g., Wang et al., 2021). Women are prone to have a green buying impulse, while men are more rational. Within the six hypothetical paths, the cohort with elevated educational attainment exhibits significance in all paths excluding the one concerning the perceived influence of information utility on intentions to make eco-friendly purchases. This suggests that individuals with higher education levels demonstrate pronounced and distinct intentions to purchase eco-friendly items. While low-income groups are important in other routes, the study findings indicate that monthly income is not significant in the path of social media marketing on eco-friendly purchasing intention, which shows that it is easier for high-income groups to avoid low-income groups to buy green products after the same exposure to info about eco-friendly items in social media. Economic theory shows that income is one of the important factors affecting consumption, so high-income groups will demonstrate a greater inclination towards purchasing green products after they possess a specific comprehension of the performance of sustainable items and other aspects. The analysis of the variable regarding time spent on social media as a moderating factor reveals that individuals who dedicate over three hours daily to social media show significance across all pathways. Conversely, those who spend less than three hours demonstrate significance in all six hypothetical pathways except for the one where perceptions of information usefulness influence intentions to make eco-friendly purchases. This underscores the considerable influence of social media usage time on individuals’ intentions to buy eco-friendly products. People who have used social media for more than 3 h are more inclined to seek out and obtain pertinent information in the realm of the internet, so their understanding and cognition of green products will be deeper, thus enhancing the individual’s eco-friendly purchase intention.

## 6 Drawbacks and future directions

SEM is used to verify the hypothesis model of eco-friendly purchase intention, and the multi-group analysis method is used to study based on basic variables, and some valuable micro-level conclusions are obtained. However, this study can still proceed with an in-depth analysis of the following aspects:

(1) Regarding data acquisition, cross-sectional data may not eliminate the possibility of causal reversal among variables. Longitudinal studies can be employed to gather data on independent, intermediate, and outcome variables at various time points, thereby enhancing our ability to discern causal relationships among variables. (2) Furthermore, despite our efforts to ensure data validity, similar to many questionnaire-based studies, it remains challenging to completely avoid subjective responses from participants, thus compromising the data’s objectivity. To address this, some advanced data-collecting methods can be integrated to acquire additional data on a larger scope. Additionally, efforts can be undertaken to increase the size of sample subjects and geographical representation of the study population, thereby increasing the generalizability of research findings and enhancing their impact. (3) This study only focuses on the consumer groups in the eastern part of China, and the study’s breadth should be widened in the future. The impact mechanisms of social media marketing on intentions toward eco-friendly purchases in different areas can be studied according to their demographic characteristics and economic resource endowments. In addition, we just analyzed the intermediary function of perceived information utility and green trust in its analysis. Future research could consider other mediating variables or even introduce the antecedent variables of social media marketing for analysis, and more mechanisms of influence deserve to be further explored.

## Data availability statement

The raw data supporting the conclusions of this article will be made available by the authors, without undue reservation.

## Ethics statement

The studies involving humans were approved by the School of Economics and Management, China University of Mining and Technology. The studies were conducted in accordance with the local legislation and institutional requirements. Written informed consent for participation in this study was provided by the participants’ legal guardians/next of kin. Written informed consent was obtained from the individual(s), and minor(s)’ legal guardian/next of kin, for the publication of any potentially identifiable images or data included in this article.

## Author contributions

MW: Writing – review & editing, Writing – original draft, Methodology, Investigation, Formal analysis, Data curation, Conceptualization. RL: Writing – review & editing, Supervision, Funding acquisition, Conceptualization.
